# Higher-order structure of polymer melt described by persistent homology

**DOI:** 10.1038/s41598-021-80975-5

**Published:** 2021-01-26

**Authors:** Yohei Shimizu, Takanori Kurokawa, Hirokazu Arai, Hitoshi Washizu

**Affiliations:** 1 Graduate School of Simulation Studies, University of Hyogo, 7-1-28 Minatojima-minamimachi, Chuo-ku, Kobe, Hyogo 650-0047 Japan; 2 JTEKT Corporation, 24-1 Kokubuhiganjo-cho, Kashiwara, Osaka 582-8588 Japan; 3 Elements Strategy Initiative for Catalysts and Batteries (ESICB), Kyoto University, 1-30 Goryo-Ohara, Nishikyo-ku, Kyoto, 615-8245 Japan

**Keywords:** Polymer characterization, Chemical physics, Computational science

## Abstract

The optimal method of the polymer Materials Informatics (MI) has not been developed because the amorphous nature of the higher-order structure affects these properties. We have now tried to develop the polymer MI’s descriptor of the higher-order structure using persistent homology as the topological method. We have experimentally studied the influence of the MD simulation cell size as the higher-order structure of the polymer on its electrical properties important for a soft material sensor or actuator device. The all-atom MD simulation of the polymer has been calculated and the obtained atomic coordinate has been analyzed by the persistent homology. The change in the higher-order structure by different cell size simulations affects the dielectric constant, although these changes are not described by a radial distribution function (RDF). On the other hand, using the 2nd order persistent diagram (PD), it was found that when the cell size is small, the island-shaped distribution become smoother as the cell size increased. There is the same tendency for the condition of change in the monomer ratio, the polymer chain length or temperature. As a result, the persistent homology may express the higher-order structure generated by the MD simulation as a descriptor of the polymer MI.

## Introduction

Materials Informatics (MI) is a technique for predicting unknown materials by learning experimental data or calculated data by computer simulation to build some prediction models. Although the number of studies of the MI of polymers is gradually growing, further research is needed in the future. The reason is that organic materials are often used as a mixture, the number of combinations is infinite, and the characteristics significantly change depending on the process such as the molding method, and the amount of teacher data is limited because many experiments are needed^1^. For simple liquids, distribution function theories can predict the structure of liquids. If the pair distribution function is known, the thermodynamic properties of the system can be obtained by a number of different theoretical routes, and they are related to the experimentally obtained structure factors^2,3^. For the polymers, on the other hand, the process affects the higher-order structure. Thus, the development of a descriptor of the higher-order structure is important for primitive problem solving.

In previous research, there are examples of machine learning using DFT data obtained from public databases and descriptors obtained by converting their molecular structures into fingerprints^4,5^. Fingerprints are expressed as binary data of a specific length based on a specific format from the molecular structure. Since fingerprints are created based on chemical formula information, the machine learning of fingerprints as descriptors cannot predict unknown higher-order structures. This is because its effect is included in the nonlinearity of the machine learning model. There is another previous study focusing on polymers and machine learning methods using PolyInfo of public database. Polymers are designed with a high probability using the fingerprints of monomers as descriptors with Bayesian inference, and predict the thermal conductivity by transfer learning^6^. In another previous research, there are also examples in which machine learning is performed using parameters, such as the presence or absence of compounding agents to be added to the polymers, and the amount of compounding as descriptors to predict the physical properties^7^. In this case, several methods have been proposed for the descriptor, and there are cases where it is described in binary format by the presence or absence of compounding agents, and there are cases when the combination amount is described as a number. However, in this method, the higher-order structure itself cannot be included in the descriptor, and the effect of compounding unknown combinations cannot be predicted.

As previous studies, since it is basically difficult to capture higher-order structures as descriptors, these substitution methods have often been done. In such situations, a topological descriptor has been considered for amorphous materials^8^. Especially, the application of persistent homology has been considered in recent years as an idea to incorporate the state of being seemingly difficult to express substances such as higher-order structures into descriptors. Persistent homology is a study called the topology of mathematics^9,10^and applied to MI as informatics tools^11^. Several analyses of amorphous materials such as metal, silica or protein, have been conducted using persistent diagrams^12–16^. There is also a study about yielding a polymer’s formation in deformation^17^.

Various studies have been conducted to understand the higher-order structures of polymers by molecular simulation. There are studies, such as how the length of the coarse-grained polymer chain or the type of bond, affects the physical properties^18,19^. Few previous studies have discussed descriptors of machine learning that express the higher-order structure of polymers. In order to apply persistent homology to the polymer MI, it is first necessary to clarify how the actual higher-order structure is expressed. Here we have focused on the simulated cell size of the molecular simulation under periodic boundary conditions. It is known that the structural behavior of liquid crystal molecules or polymer nano-fibers changes depending on the cell size even under periodic boundary conditions^20,21^. It is also known that such a difference in the cell size causes a difference in the molecular momentum^22^.

## Results

**Figure 1:**
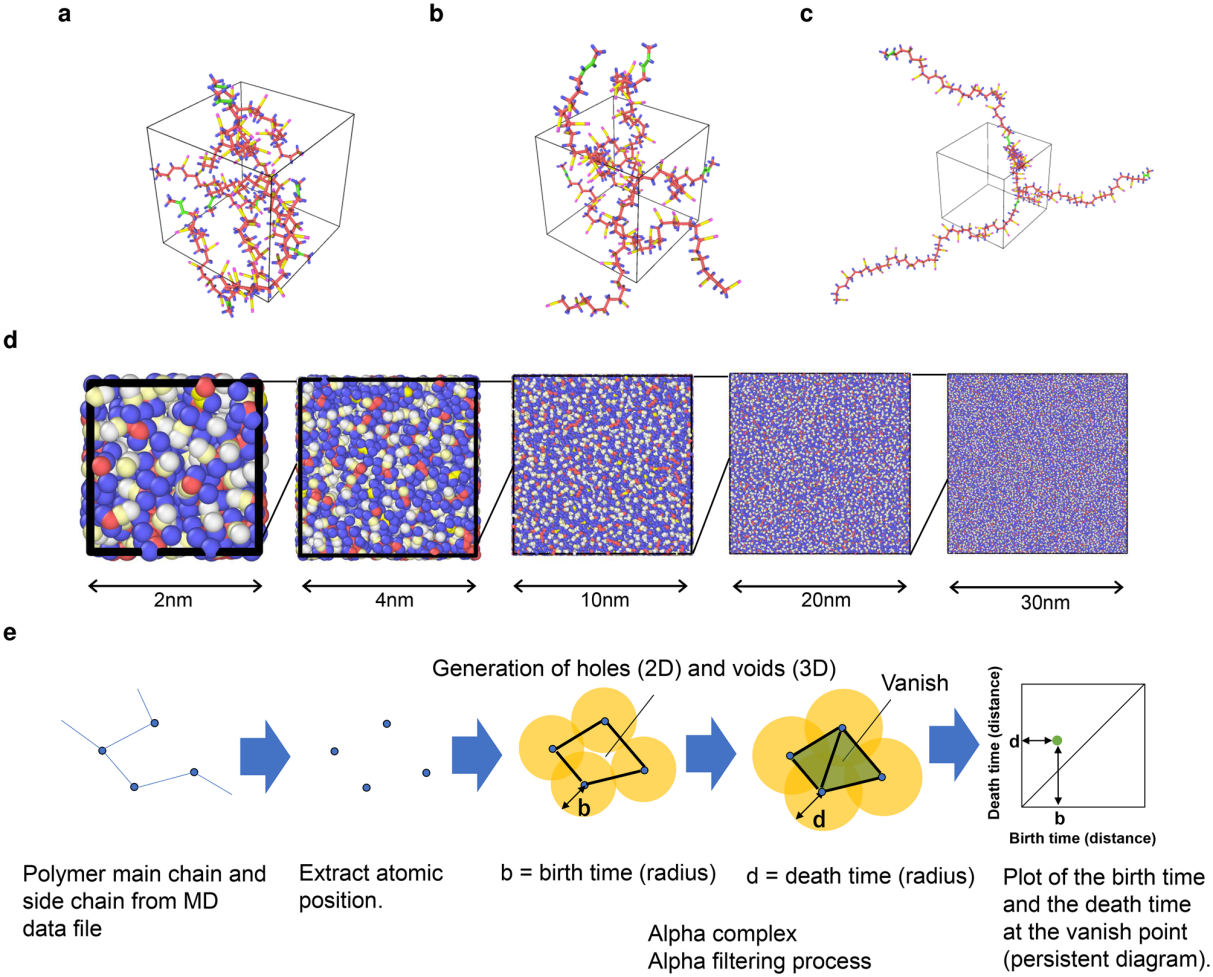
Polymer image for the simulation cell size of 2 nm in sample 1 and Conceptual diagram of persistent homology. These all atom coordinates are used for the persitent diagram computation. **(a)** 10mer of polymer chain length. The polymer does not stick out of the cubic cell. **(b)** 20 mer of polymer chain length. The polymer sticks out of the cubic cell. **(c)** 40 mer of polymer chain length. The polymer significantly sticks out of the cubic cell. **(d)** The difference in the higher-order structure change for each simulation cell size (2 nm to 30 nm) of sample 1 with different colors as different atoms in the equilibrium condition. There are abstract changes but not directly mentioned. **(e)** Method to obtain a persistent diagram (PD) using alpha comlex of each atom of a polymer from a data file of MD data. It is shown that the birth time becomes longer as the plot of the PD goes to the upper right, and it has a large structure. The farther the plot is from the diagonal line, the greater the difference between the birth time and death time, indicating a stable structure that does not easily disappear.

**Figure 2:**
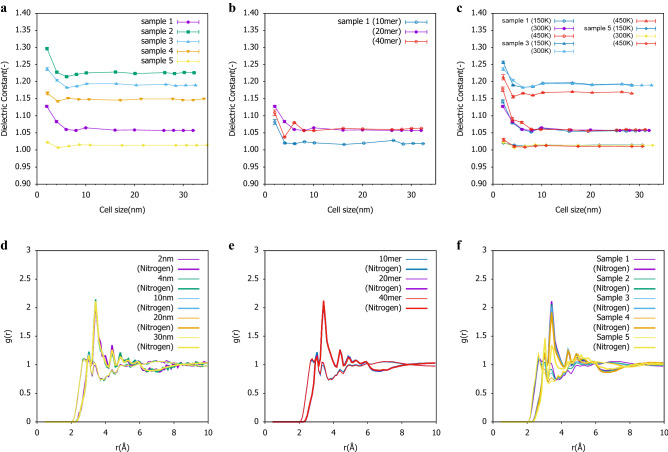
The change in dielectric constant (DC) and the radial distribution function (RDF) for the simulation cell size. Error bars in DC represent $\pm 3\sigma$. **(a)** DC effect of monomer ratio. **(b)** DC effect of polymer chain length in sample 1. **(c)** DC effect of temperature in samples 1,2 and 5. **(d)** RDF effect of simulation cell size in sample 1. The thin line is the total RDF, and the thick line is the total RDF for Nitrogen in the side chain. Same as below. **(e)** RDF effect of polymer chain length in sample 1 and 20nm cell size. **(f)** RDF effect of monomer ratio in sample 1 and 20nm cell size.

We consider the dielectric constant of polar polymer because this property is important for a soft material sensor or actuator device like a dielectric elastomer. Considering NBR (Acrylonitrile-Butadien) rubber, a randomly polymerized polymer composed of two kinds of monomers of acrylonitrile and butadiene is prepared under the conditions shown in Table 1, Fig. 1a–d. Despite the difference in the monomer ratio, as the cell size change from about 2 nm to 32 nm, the relative dielectric constant changes and equilibrates at about 20 nm despite the same polymer (Fig. 2a). The same tendency is observed when the polymer chain length is changed, but the dielectric constant after equilibration is different depending on the ratio (Fig. 2b). This is considered to indicate that the polymer chain length that equilibrates the dielectric constant is 20 mer. The same tendency is observed when the temperature is changed, but it was found that the variation in the monomer ratio for a smaller cell size is different(Fig. 2c). This is considered to be related to the glass transition temperature. At this time, no clear difference is observed in the overall radial distribution function (RDF) due to changes in the cell size shown in Fig. 2d^23^. Figure 2e,f show the differences in the RDFs when the polymer chain length and the monomer ratio were changed for a cell size of 20 nm with a stable dielectric constant. The RDF does not change when the polymer chain length was changed. On the other hand, it was confirmed that when the monomer ratio is changed, the peak corresponding to the monomer has been changed. The same tendency is observed not only in the overall RDF but also in the nitrogen in the side chain of the NBR polymer and the RDF focusing on the whole. Based on these results, it was confirmed that the relative dielectric constant changed not due to the primary structure but due to the higher-order structure^24^. Because this overall movement cannot be detected by the RDF, it is thought that it affects not the primary structure but the higher-order structure.

**Table 1:** Polymer composition and equilibrium temperature conditions.

Sample	Side length of cubic cell	Acrylonitrile	Butadiene	Polymer chain	Equilibrium
name	before expansion (nm)	ratio (mol%)	ratio (mol%)	length (mer)	temperature (K)
Sample 1	2.0038	95	5	10, 20, 40	150 ,300, 450
Sample 2	2.0488	70	30	20	300
Sample 3	2.1011	50	50	10, 20, 40	150, 300, 450
Sample 4	2.1445	30	70	20	300
Sample 5	2.1690	5	95	10, 20, 40	150 ,300, 450

The 0th order, 1st order, and 2nd order for the persistent diagram (PD) using alpha complex are calculated using the method shown in Fig. 1e. All the results of sample 1 are shown in Fig. S1. These diagrams represent the bonding state, the hole, and the air gap respectively. It is calculated for each cell size using the coordinates of all atoms in the final step of MD calculation. In order to compare PDs with the same frequency as possible, the cell size of 2, 4, 10 nm is the same as 20 nm by duplicating all the atomic coordinates of the final step. Above RDF calculation targeted 10 angstroms around the atom. Because the unit of birth time and death time in the PD is the square of angstrom, the region less than 5 angstroms around the atom is calculated in this time. As a result, a remarkable difference is found especially in the 2nd order voids, and the PD shows a discrete tendency with about 17 aggregations for a cell size of 2 nm in sample 1 (Fig. 31-a–1-e). For the cell size of 4 nm, these aggregation with the cell size of 2 nm disperse , and a new aggregation is obtained in the outer (upper) area. As the cell size increases in this way, between each aggregation has dispersed evenly. A PD without obvious aggregation is obtained for the cell size of 20 nm and the shape has not changed in greater than this cell size. This indicates that a number of stable structures have appeared, and is considered to abstractly represent that the distance between the polymers has increased. The results are consistent with the change in the dielectric constant.

**Figure 3:**
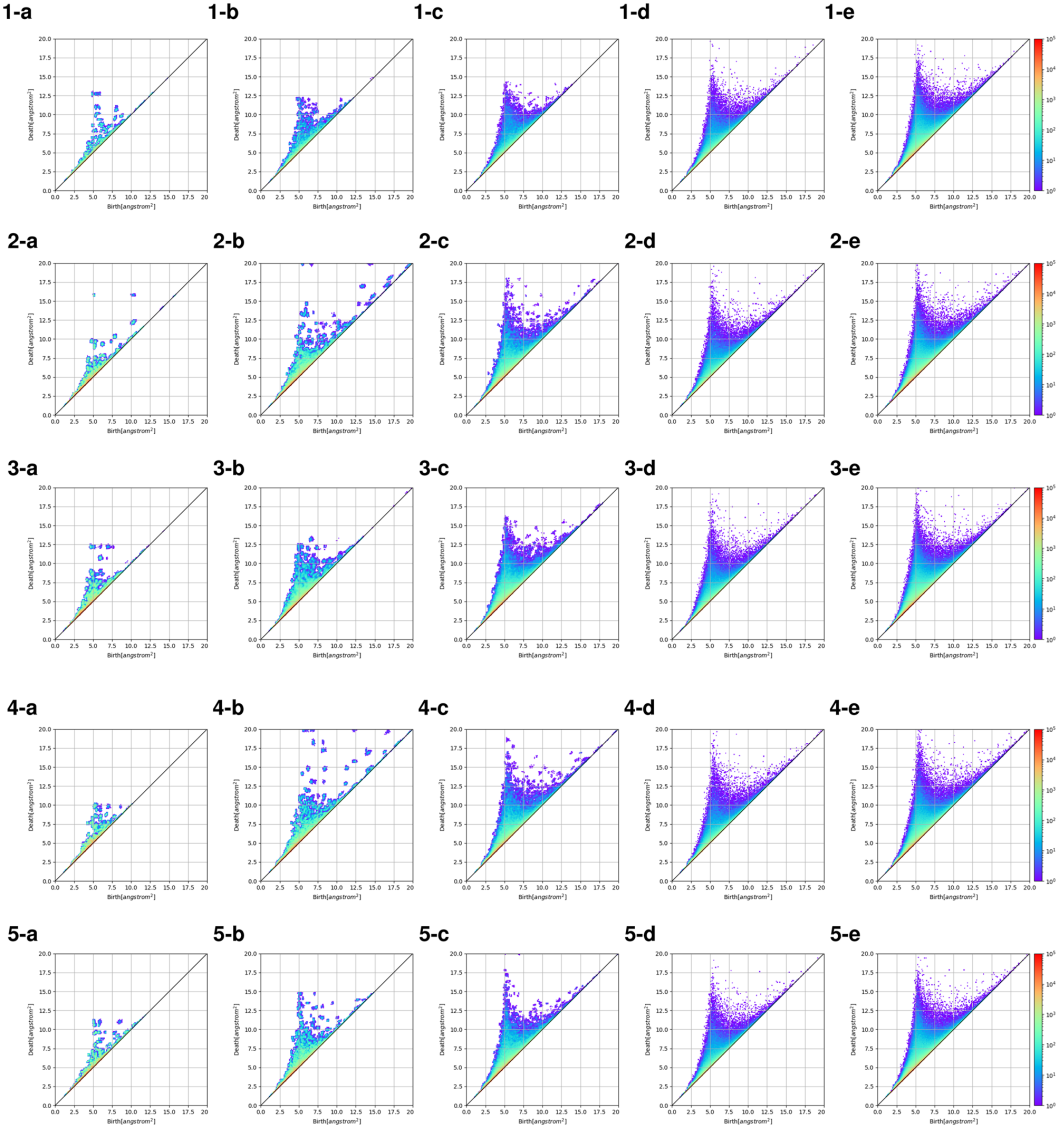
The change in the 2nd-order persistent diagrams with a hydrogen coordinate in each simulated cell size (2 nm to 30 nm). **(a)** 2 nm expand to 20 nm, **(b)** 4 nm expand to 20 nm, **(c)** 10 nm expand to 30 nm, **(d)** 20 nm, **(e)** 30 nm. Line 1 is sample 1. Line 2 is sample 2. Line 3 is sample 5. Line 4 is 10 mer in sample 1. Line 5 is 40 mer in sample 1.

**Figure 4:**
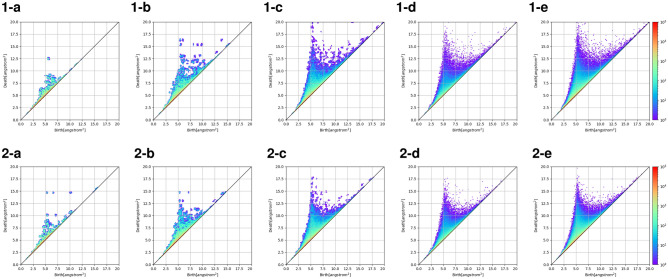
The change in the 2nd-order persistent diagrams with a hydrogen coordinate in each simulated cell size (2 nm to 30 nm). **(a)** 2 nm expand to 20 nm , **(b)** 4 nm expand to 20 nm, **(c)** 10 nm expand to 30 nm, **(d)** 20 nm, **(e)** 30 nm. Line1 is 150 K in sample 1. Line 2 is 450 K in sample 1.

The same tendency is observed when the monomer ratio (Fig. 3 2-a–2-e, 3-a–3-e), the polymer chain length (Fig. 3 4-a–4-e, 5-a–5-e) and the temperature (Fig. 41-a–1-e, 2-a–2-e) are changed. It is found that when the monomer ratio is changed, the aggregation of PD is different for the small cell sizes, and a stable structure appeared depending on the ratio. Regarding the difference in the monomer ratio, the greater the butadiene ratio has the large aggregation for a cell size of 2 nm (Fig. 31-a,2-a,3-a). This indicates that there is a larger and the stable structure . This is considered to represent the ease of rotation of the polymer. It is considered that suppression of the polymer rotation is also reflected in the large change in cell the size distribution. A detailed examination of the PD when the polymer chain length is changed revealed that the island distribution of the 10 mer persistent map has appeared in a large range for a cell size of 4 nm (Fig. 31-b,2-b,3-b). No such distribution is observed for the 20 mer and 40 mer, suggesting a difference in the structure depending on the cell size. The change in temperature is a plot of the change in the dielectric constant above and below the glass transition temperature. It is found that these changes clearly appeared when the cell size is 2 nm or 4 nm (Fig. 31-a,b, Fig. 41-a,b,2-a,b). This is considered to represent that the rotation of the polymer is suppressed by the temperature and the possible shapes are limited. In this way, it was found that the difference in polymers can be clearly extracted by using the PD.

Furthermore, some results are shown to confirm that the 2nd order PD represents the higher order structure. It is the same tendency for the 2nd order diagram with the hydrogen coordinate and without that in sample 1 (Fig. S2). It was confirmed that the PD represents the polymer skeleton independent of the presence or absence of hydrogen. Also, there is no effect on the 2nd order persistent diagram due to expanding for equalizing the frequency of the histograms. These results before expanding are shown in Fig. S3. It was confirmed that there are differences in the frequency although the overall trends do not change. Since the frequency influences the accuracy of the vectorization after this method, the cell size is unified as much as possible.

## Discussion

**Figure 5:**
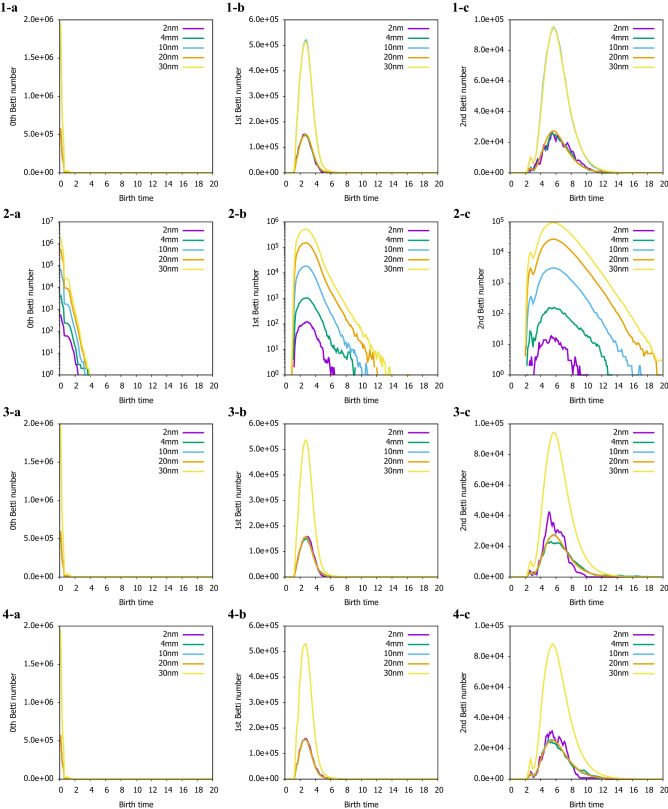
The change in the persistent betti numbers (PBNs) in sample 1. (1) 300 K and 20 mer, (2) 300 K and 20 mer that PD of cellsize 2, 4 and 10 nm without cellsize expanding, (3) 300K and 10 mer, (4) 150 K and 20 mer. **(a)** 0th PBNs, **(b)** 1st PBN, **(c)** 2nd PBNs.

In order to compare the changes in PD with cell size more quantitatively, the number of the persistent betti numbers^25,26^ (PBNs) from the 0th to 2nd order is calculated shown in Fig. 5. There is no change in the distribution of 0th order and 1st-order PBNs depending on the cell size shown in Fig. 51-a–1-c. The cell size of 2, 4, and 10 nm is adjusted to have the same frequency as the cell size of 20 nm, and the frequency is different only for the cell size of 30 nm, but the tendency is the same. It is found that the distribution of secondary PBNs varied at cell sizes of 2 nm and 4 nm, where clear aggregation is observed in PD, and that as the cell size increased, the peak positions remain the same and changed to a gentle distribution. Fig. 52-a–2-c are PBNs when the frequency of cell sizes 2, 4, and 10 nm is not adjusted. Since the frequency varies greatly depending on the cell size, the vertical axis is displayed here on a log scale. There was no change in the tendency of the number of 0th order and 1st order persistent Betti numbers, but the distribution of cell sizes of 2 and 4 nm was different in the 2nd order persistent Betti numbers as in Fig. 51-c. From this, it is considered that the result is not affected by adjusting the frequency. Fig. 53-a–3-c have a polymer chain length of 10 mer, and Fig. 54-a–4-c have a temperature of 150 K. In each case, there are clear variations in the cell size of the 2nd order PBNs of 2, 4 nm. In particular, the variation in Fig. 53-c is larger than in other conditions, suggesting that the relationship between cell size and polymer chain length contributes significantly.

**Figure 6:**
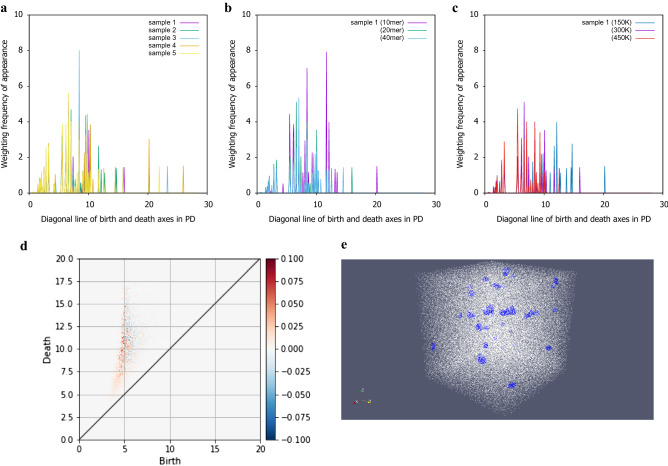
The change of the Vectrized persistent diagram (PD) and PD site and real space location that contribute to monomer ratio. **(a–c) ** is a weighting frequency of appearance vectorized in cell size of 20 nm. Each spectrum is different. **(a)** Effect of monomer ratio. **(b)** Effect of polymer chain length. **(c)** Effect of temperature. **(d)** The site where the monomer ratio contributes in the persistent diagram using PCA in cell size of 20 nm. This graph shows the 1st principal component, where red is positive and blue is negative. The red part is the part that contributes to the monomer ratio. **(e)** Inverse analysis results of Delaunay complex distributions in real space affect the monomer ratio.

Differences due to monomer ratio, polymer chain length, and temperature in the PDs with cell sizes of 20 nm and above are not clear. Therefore, in order to qualitatively compare the persistent diagrams for the cell size of 20 nm, the shapes of the vectors weighted according to the distance from the diagonal are compared. A clear difference is observed in the vector due to the difference in the monomer ratio, polymer chain length and temperature for the cell size of 20 nm (Fig. 6a–c). In order to clarify the position where the monomer ratio is expressed in the PD for the cell size of 20 nm, a dimension reduction is performed using PCA from the vectorized persistent diagram and the contributing sites are displayed (Fig. 6d). It was found that the cumulative contribution rate of PCA is almost 1 in 4 dimensions. Fig. 6d shows the positive and negative contributions of the first principal component with a contribution ratio of 0.3, where red is positive and blue is negative. It was found that the structure of the red part in the PD has a significant influence on the monomer ratio. An inverse analysis that affected the monomer ratio is shown in Fig. 6e. It was found that the voids in the uniform structure were dispersed.

**Figure 7:**
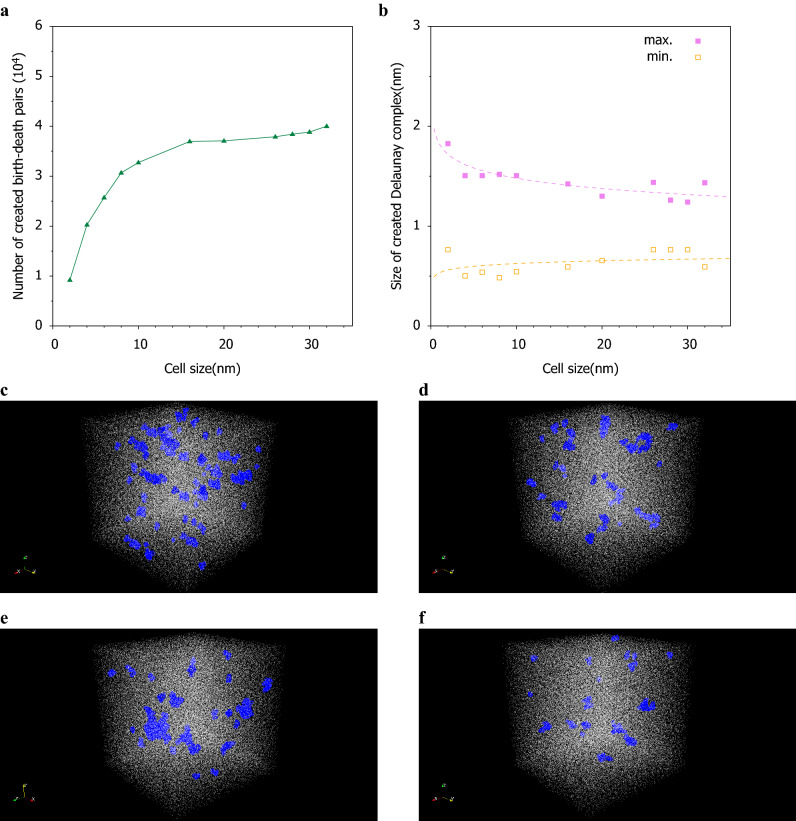
The change of Delaunay complexes and inverse analysis in Sample 1 **(a)** Relationship between the cell size and created birth-death pairs. **(b)** Relationship beween the cell size and maximum or minimum size of created Delaunay complexes. Inverse analysis results of Delaunay complexes distributions in real space expanded to 20 nm cell. **(c)** Inverse analysis results of Delaunay complexes distributions in real space in 2 nm cell expanded to 20 nm cell, **(d)** Similar 4 nm expanded to 20 nm, **(e)** Similar 10 nm expanded to 20 nm, **(f)** Similar 20 nm without expanded.

To investigate the relationship between the cell size and polymer length, the number of newly appearing structures in the PD of each cell size before equilibration of the dielectric constant was determined for a quantitative comparison. Using the vectorized PD, the difference from each cell size was obtained. The number of birth-death pairs of sample 1 was extracted and accumulated for each cell size which is shown in Fig.S4. The results, which are shown in Fig. 7a, also indicate equilibration for the cell size of 20 nm. The cell size of 20 nm is about 5 times the polymer chain length, and it is considered that the new higher-order structure is stable at this size in sample 1. Therefore, by using persistent homology, it is possible to clarify the change not due to the primary structure but by the higher-order structure.

An inverse analysis was performed to discuss the physical meaning of the persistent diagram in sample 1. The maximum and minimum sizes of the birth-death pairs are estimated by these vectorized persistent diagrams. The difference between the maximum and the minimum sizes is high when the cell size is small, and the size of the birth-death pairs varies, but when the cell size is large, the difference becomes small and the size of these pairs becomes uniform which is shown in Fig. 7b. In order to confirm the distribution of these pairs, an inverse analysis was performed using the optimal volume method of Homcloud software, and 2% are randomly arranged in real space as Delaunay complexes. Here, the cell sizes of 2, 4, 10 and 20 nm are unified and compared to the cell size of 20 nm that is balanced by the persistent diagram which is shown in Fig. 7c to f. The size of the Delaunay complex is larger than the size between the atoms, and such voids do not actually exist. It is considered that this difference in the virtual void structure affects the higher-order structure. The Delaunay complex of the virtual void is evenly distributed over all the cell sizes. It can also be seen that the size distribution varies when the cell size is small, but becomes uniform when the cell size increases. For comparison, an image of the size of the Delaunay complex at the vertex of the persistent diagram before expansion is shown in Fig.S5. It can be confirmed that even with the cell size before expansion, the size of the Delaunay complex relative to the whole is small.

It can be considered that as the all-atom MD simulation cell size increases, there is no restriction on the structure of the polymer and it can also freely move in spite of the periodic boundary condition. In the case of a cell size smaller than the polymer chain length, the orientation of the individual polymer oriented by the electric field is preserved by the periodic boundary condition, so that the polymer is easily polarized. On the other hand, in the case of a cell size larger than the polymer chain length, the direction of the polymer completed in the cell is not affected by the periodic boundary condition, but is affected by the surrounding polymer which is shown in Fig.S6.

This is considered to indicate a higher-order structure. It is also considered that the use of the persistent homology indicates the possibility of arranging the higher-order structures as a difference in the abstract polymer skeleton structures. It is expressed in real space by the variation and distribution of the size of the Delaunay complex of the virtual void structure. Furthermore, they are represented as differences in the histograms in the persistent diagram. By observing the change in the persistent diagram, it may be possible to distinguish and understand the monomer ratio, polymer chain length, and temperature. We consider that the PD has physical meaning. As the cell size increases, the size of the virtual voids composed of the polymer skeleton becomes smaller and uniform, and it is possible that this may be associated with changes in the dielectric constant. These results suggest that the PD of the polymer can apply machine learning as a descriptor in the polymer MI with a physical meaning. In the polymer science, a much larger structure in the mesoscopic level, such as the lamellar structures by the repetition of amorphous and crystal structure in polyethylene^27^, should be treated in the coarse-grained simulation, and considered in another mathematical framework.

## Methods

The NBR polymer shown in Table 1 is randomly copolymerized from 5 to 95 mol% acrylonitrile monomers and 95 mol% to 9 mol% butadiene monomers for becoming 10 mer, 20 mer or 40 mer. The polymer chain length is about 2 nm to 8 nm. In order to adjust the number of atoms, 8 polymers at 10 mer, 4 polymers at 20 mer, and 2 polymers at 40 mer are stored in a cubic cell with an initial density of 0.797. The periodic boundary conditions are then applied. One side of the cell size is about 2 nm. The atomic charge is obtained from the AM1-BCC model^28^. Next, the cell size is increased from 2 nm to 32 nm while maintaining the specific gravity by replicating. At this time, the number of polymers increases from 4 to 16,384 at 20 mer. We evaluated between the cell size and polymer chain length as a higher-order structure. After modeling, the MD simulation is executed. The Dreiding force^29^ field is used to calculate the intra-molecular interactions. The initial equilibration conditions are 1ns with the NPT ensemble, 500 K, and 1000 atm. After this step, the density is about 0.98. This process is empirically a necessary step to generate and equilibrate higher-order structures in a short time. In the next step, equilibrium calculations for annealing to the target temperature are performed. The temperature is set at 300 K, and 1ns with the NVT ensemble. In order to confirm the effect of the glass transition temperature for some polymers, equilibrium calculations were performed at temperatures of 150 K and 450 K. After these initial equilibration steps, an electric field is next applied to the simulation cell. A DC electric field of $1.0\times 10^{-3}$ V/nm is applied in the Y-axis direction under the conditions of the NVT ensemble, 300 K, 150 K or 450 K, and after the 1 ns calculations, it was confirmed that the kinetic energy is stable and the RDF does ‘not’ change. Using the polarization obtained by adding the dipole moment in the X, Y, and Z directions of each atom, the dielectric constant for each cell size is calculated according to (1)^30^.

1 $$\begin{aligned} \epsilon _r=\frac{P}{\epsilon _0E}+1 \end{aligned}$$

where $\epsilon _r$ is the dielectric constant, $\epsilon _0$ is the dielectric constant under the vacuum condition, *E* is the degree of the electric field, and *P* is the polarization. The radial distribution function *g*(*x*) is output by dividing the range of 0 to 1 nm into 1000. The parallel code LAMMPS (Version Lammps-22Aug18, http://lammps.sandia.gov) is used to implement the MD simulation. Winmostar (Winmostar Version 8, X-Ability Co., Ltd., Tokyo, Japan, 2017) is used to operate these modelings.

The second step is the calculation of the persistent homology. Persistent homology is a method of topology. Here, we consider the alpha complex and alpha filtration. Increase the circle assigned to all atomic coordinates of the polymer, and the time (radius) surrounded by the circle (sphere in the case of 3D) is defined as the birth time. The circle (sphere) is evenly enlarged, and the time (radius) when the part surrounded by the circle disappears is defined as the death time. A plot of all combinations of the birth time and death time is called a persistent diagram (PD). It is shown that the birth time becomes longer as the plot of the PD goes to the upper right, and it has a large structure. The farther the plot is from the diagonal line, the greater the difference between the birth time and death time, indicating a stable structure that does not easily disappear. In this research, the position coordinates of all the atoms are extracted from the obtained MD data file results. Using the point cloud method of the Homcloud software (Version Homcloud 2.8.1, http://www.wpi-aimr.tohoku.ac.jp/hiraoka_labo/homcloud-english.html), we calculated the 0th order, 1st order, and 2nd order persistent pairs of the birth-time and death-time and output them as a histogram called the PD. The number of divisions is 256. The resulting histogram represents the number of pairs. The total number of pairs for each cell size is obtained. For a cell size smaller than 20 nm, the snapshots are replicated to fill the larger cell size, and the coordinates are acquired after expanding to 20 nm, 30 nm or 32 nm because the same frequency is obtained as much as possible. Furthermore, in order to confirm whether the polymer skeleton is represented, the same calculation is performed using coordinates without hydrogen atoms^31^. The number of persistent betti numbers (PBNs) is calculated by counting the number of pairs for which $death \; time > a + 0.1$ when $birth \; time \leqq a$ (where a changes from 0 to 20 in 0.1 increments) from each birth death pair.

Next, the vectorization processing was excecuted^32^ for the the inverse analysis. The gradation is 256 on the $20 \times 20$ PDs. The standard deviation is 0.002. The weight conditions are $\hbox {tan}^{-1}$ ($0.01 \times { l}^3$), where ${ l}$ is $lifetime ( = birth - death)$. After comparing the shapes of the vectors obtained under the conditions of the monomer ratio difference, a standardization is performed and the dimension is reduced to 4 by Principal Component Analysis (PCA). The obtained average vector has been inversely analyzed, and the regions where each condition contributed in the PD are illustrated. Next, the difference vector between adjacent cell sizes is inversely transformed into a PD. This makes it possible to extract the area of the histogram that appears as the cell size increases. A histogram is displayed only in the frequency region of 0.3 or more. In the extracted PD, the points farthest and closest from the diagonal of the birth-time and the death-time are obtained. The obtained square roots of ${ l}$ are defined as the maximum and minimum Delaunay complex sizes. Finally, 2% of the extracted pairs from each extracted diagram are randomly selected from the obtained distribution, and an inverse analysis is performed using the optimal volume method^33,34^. For each obtained Delaunay complex, its shape and position in the coordinate system expanded to 20 nm are confirmed.

## Supplementary information

Supplementary Figures.

## Data Availability

Details of the simulations are available within the article and the supplementary information. All data supporting the findings of this study are available from the authors upon reasonable request.

## References

[1] Audus D. J, de Pablo J. J. (2017). Polymer informatics: Opportunities and challenges. ACS Macro Lett..

[2] Hansen J.-P, McDonald I. R (2013). Theory of simple liquids. Theory of Simple Liquids.

[3] Kirkwood JG, Buff FP (1951). The statistical mechanical theory of solutions. I. J. Chem. Phys..

[4] von Lilienfeld OA, Ramakrishnan R, Rupp M, Knoll A (2015). Fourier series of atomic radial distribution functions: A molecular fingerprint for machine learning models of quantum chemical properties. Int. J. Quantum Chem..

[5] Mannodi-Kanakkithodi A (2018). Scoping the polymer genome: A roadmap for rational polymer dielectrics design and beyond. Mater. Today.

[6] Stephen W (2019). Machine-learning-assisted discovery of polymers with high thermal conductivity using a molecular design algorithm. npj Comput. Mater..

[7] Ikeda Y, Okuyama M, Nakazawa Y, Oshiyama T (2019). Materials informatics approach to predictive models for elastic modulus of polymer composites. Konica Minolta Tech. Rep..

[8] Lee, Y. *et al.* Quantifiying similarity of pore-geometry in nanoporous materials. *Nat. Commun.* **8**. 10.1038/ncomms15396 (2017). 10.1038/ncomms15396 PMC5457500 28534490

[9] Edelsbrunner, H. & Harer, J. Persistent homology—A survey. *Ser. Contemp. Sppl. Math.* **453**. 10.1090/conm/453/08802 (2008).

[10] Zomorodian A, Carlsson G (2005). Computing persistent homology. Discrete Comput. Geom..

[11] Tanaka I (2018). Nanoinformatics, 75–95.

[12] Takiyama A, Teramoto T, Suzuki H, Yamashiro K, Tanaka S (2017). Persistent homology index as a robust quantitative measure of immunohistochemical scoring. Sci. Rep..

[13] Duman A, Yilbas B, Pirim H, Ali H (2017). Texture analysis of hydrophobic polycarbonate and polydimethylsiloxane surfaces via persistent homology. Coatings.

[14] Murakami M (2019). Ultrahigh-pressure form of $$ \text{Si}\text{O}_{2}$$ glass with dense pyrite-type crystalline homology. Phys. Rev. B.

[15] Kimura M, Obayashi I, Takeichi Y, Murano R, Hiraoka Y (2018). Non-empirical identification of trigger sites in heterogeneous processes using persistent homology. Sci. Rep..

[16] Gameiro M (2015). A topological measurement of protein compressibility. Japan J. Indust. Appl. Math..

[17] Ichinomiya T, Obayashi I, Hiraoka Y (2017). Persistent homology analysis of craze formation. Phys. Rev. E.

[18] Daivis P, Matin M, Todd B (2003). Nonlinear shear and elongational rheology of model polymer melts by non-equilibrium molecular dynamics. J. Non-Newtonian Fluid Mech..

[19] Hosono N, Masubuchi Y, Furukawa H, Watanabe T (2007). A molecular dynamics simulation study on polymer networks of end-linked flexible or rigid chains. J. Chem. Phys..

[20] Mima T, Narumi T, Kameoka S, Yasuoka K (2008). Cell size dependence of orientational order of uniaxial liquid crystals in flat slit. Mol. Simul..

[21] Curgul S, Van Vliet KJ, Rutledge GC (2007). Molecular dynamics simulation of size-dependent structural and thermal properties of polymer nanofibers. Macromolecules.

[22] Washizu H, Hyodo S-A, Ohmori T, Nishino N, Suzuki A (2014). Macroscopic no-slip boundary condition confirmed in full atomistic simulation of oil film. Tribol. Online.

[23] Onodera Y (2019). Understanding diffraction patterns of glassy, liquid and amorphous materials via persistent homology analyses. J. Ceram. Soc. Jpn..

[24] Han, Y. & Elliott, J. Molecular dynamics simulations of the elastic properties of polymer/carbon nanotube composites. *Comput. Mater. Sci.* **315–323**, 10.1016/j.commatsci.2006.06.011 (2007).

[25] Meng Z, Anand DV, Lu Y, Wu J, Xia K (2020). Weighted persistent homology for biomolecular data analysis. Sci. Rep..

[26] Anand DV, Meng Z, Xia K, Mu Y (2020). Weighted persistent homology for osmolyte molecular aggregation and hydrogen-bonding network analysis. Sci. Rep..

[27] Higuchi Y (2019). Stress transmitters at the molecular level in the deformation and fracture processes of the lamellar structure of polyethylene via coarse-grained molecular dynamics simulations. Macromolecules.

[28] Jakalian, A., Jack, D. B. & Bayly, C. I. Fast, efficient generation of high-quality atomic charges. am1-bcc model: II. Parameterization and validation. *J. Comput. Chem.* **23**, 1623–1641, 10.1002/jcc.10128 (2002). https://onlinelibrary.wiley.com/doi/pdf/10.1002/jcc.10128. 10.1002/jcc.10128 12395429

[29] Mayo SL, Olafson BD, Goddard WA (1990). Dreiding: A generic force field for molecular simulations. J. Phys. Chem..

[30] Taotao H (2018). The predicted dielectric constant of an amorphous pvdf changing with temperature by molecular dynamics simulations. Int. J. Electrochem. Sci..

[31] Verovek, S. K. & Mashaghi, A. Extended topological persistence and contact arrangements in folded linear molecules. *Front. Appl. Math. Stat.* **2**, 10.3389/fams.2016.00006 (2016).

[32] Obayashi I, Hiraoka Y, Kimura M (2018). Persistence diagrams with linear machine learning models. J Appl. Comput. Topol..

[33] Gameiro M, Hiraoka Y, Obayashi I (2016). Continuation of point clouds via persistence diagrams. Physica D: Nonlinear Phenom..

[34] Obayashi I (2018). Volume-optimal cycle: Tightest representative cycle of a generator in persistent homology. SIAM J. Appl. Algebra Geom..

