# Supplementary material for: Higher-order structure of polymer melt described by persistent homology
Source: Sci Rep. 2021 Jan 26;11:2274. doi: 10.1038/s41598-021-80975-5 (PMC7838420; doi:10.1038/s41598-021-80975-5)
Supplement: Supplementary file 1 — Supplementary Figures. [file 41598_2021_80975_MOESM1_ESM.pdf]

Supplementary Information :
Higher-Order Structure of Polymer Melt Described by Persistent Homology

Yohei SHIMIZU[1,2], Takanori KUROKAWA[2], Hirokazu ARAI[2], and Hitoshi WASHIZU[1,3,*]

[1]Graduate School of Simulation Studies, University of Hyogo, 7-1-28 Minatojima-minamimachi, Chuo-ku, Kobe, Hyogo 650-0047, Japan. Fax: +81 (0)78 303 1975; Tel: +81 (0)78 303 1975
[2]JTEKT Corporation, 24-1 Kokubuhiganjo-cho, Kashiwara-shi, Osaka 582-8588, Japan.
[3]Elements Strategy Initiative for Catalysts and Batteries (ESICB), Kyoto University, 1-30 Goryo-Ohara, Nishikyo-ku, Kyoto 615-8245, Japan.
[*]Corresponding Author; E-mail: h@washizu.org

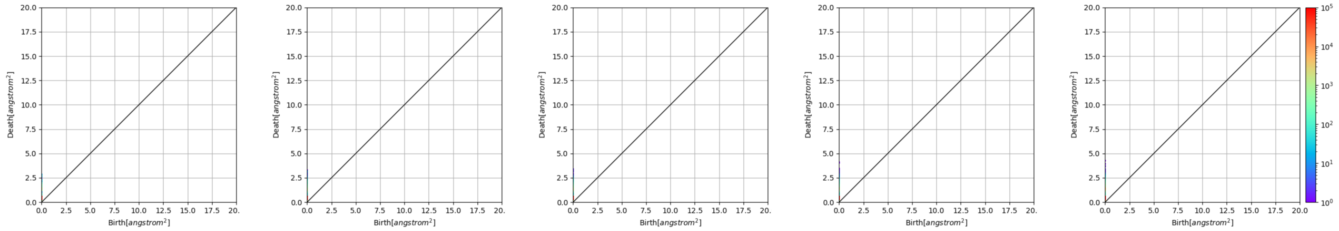

(a) 2nm, 0th order (b) 4nm, 0th order (c) 10nm, 0th order (d) 20nm, 0th order (e) 30nm, 0th order

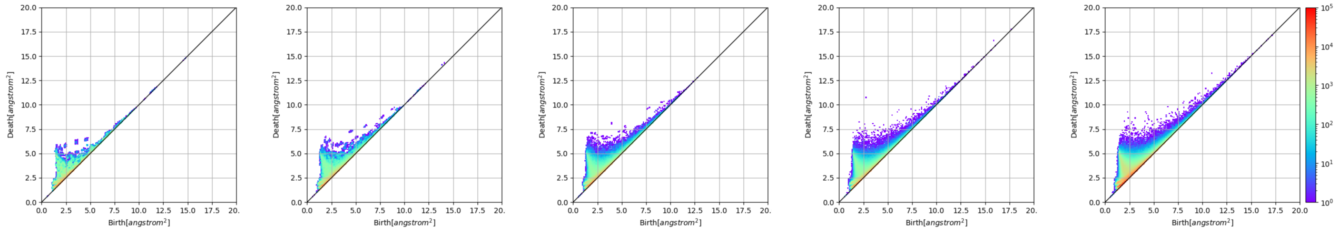

(f) 2nm, 1th order (g) 4nm, 1th order (h) 10nm, 1th order (i) 20nm, 1th order (j) 30nm, 1th order

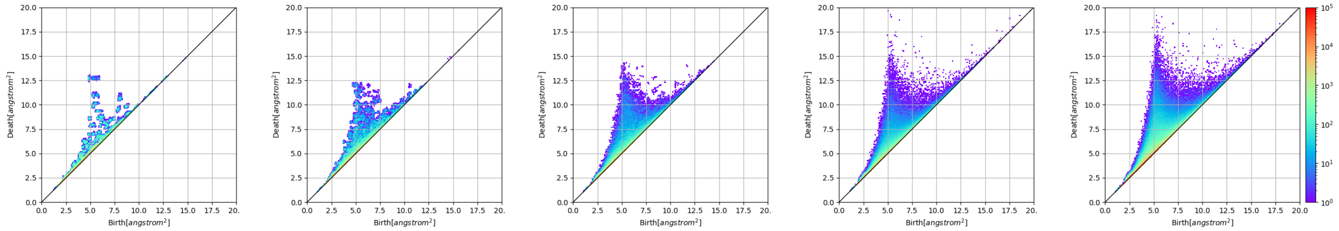

(k) 2nm, 2th order (l) 4nm, 2th order (m) 10nm, 2th order (n) 20nm, 2th order (o) 30nm, 2th order

Figure S1: The change in the 0th to 2nd order persistent diagrams for each simulation cell size in sample 1. In the 2nd order persistent diagrams, there is an island shape in the 3nm cell size, and the shape continiously changed as the cell size increased. 2nm expand to 20nm, 4nm expand to 20nm, 10nm expand to 30nm.

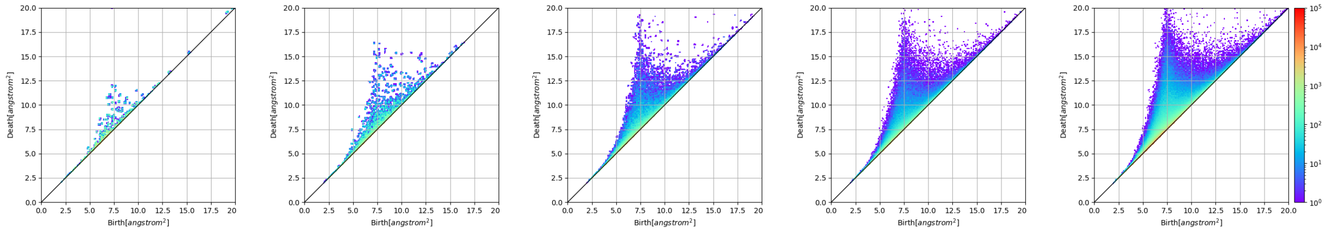

(a) 2nm (b) 4nm (c) 10nm (d) 20nm (e) 30nm

Figure S2: The change in the 2nd order persistent diagrams without a hydrogen coordinate in each simulated cell size in sample 1. 2nm expand to 20nm, 4nm expand to 20nm, 10nm expand to 30nm. The whole shape is not different from the persistent diagrams with the hydrogen coordinate.

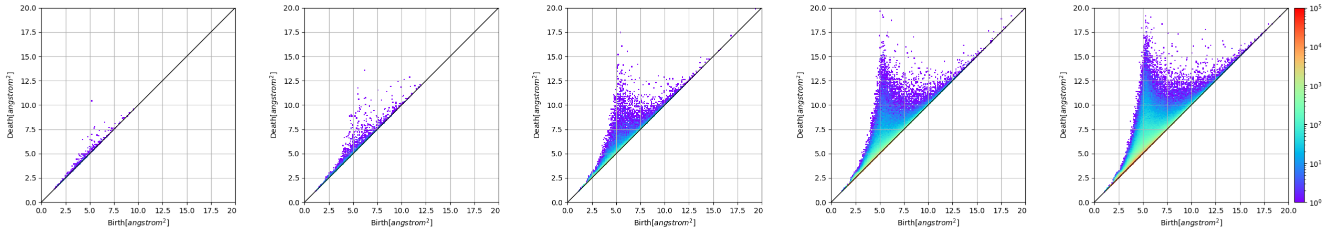

(a) 2nm (b) 4nm (c) 10nm (d) 20nm (e) 30nm

Figure S3: The change in the 2nd order persistent diagrams before expanding cell size in sample 1. No change in trend compared to the expanded persistent diagram.

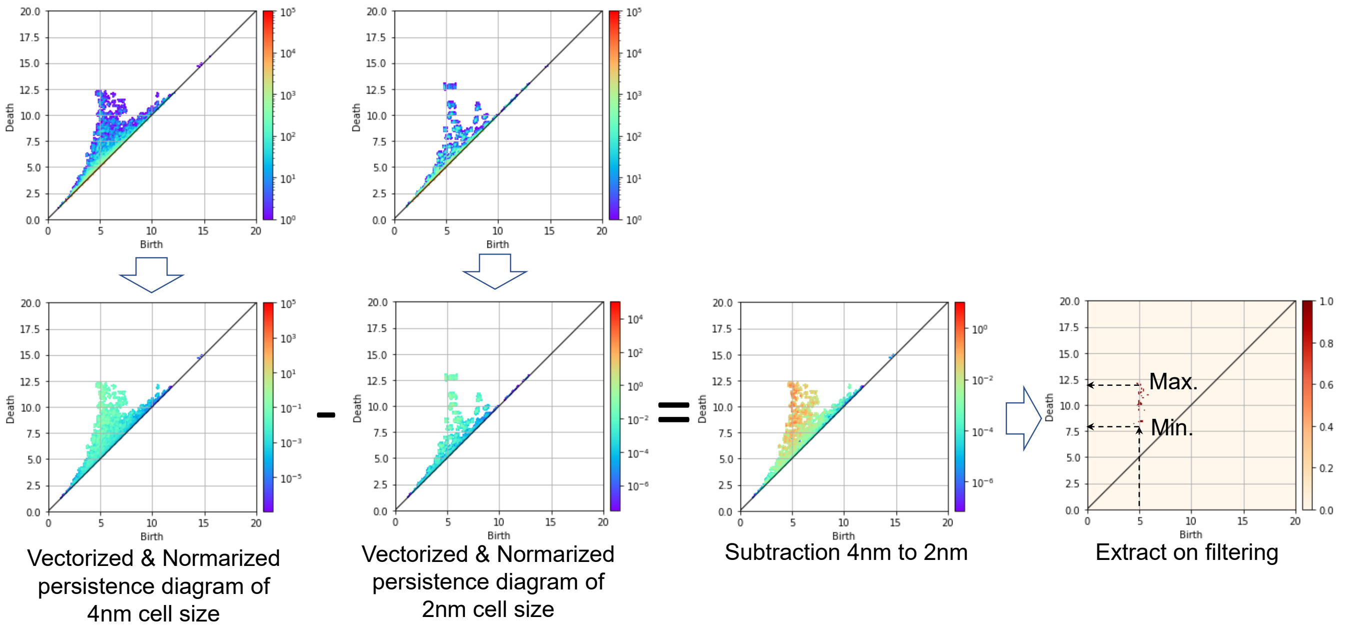

Figure S4: The example of vectorized persistent homology and extract distribution of the difference and estimation of the vertual void structure size.

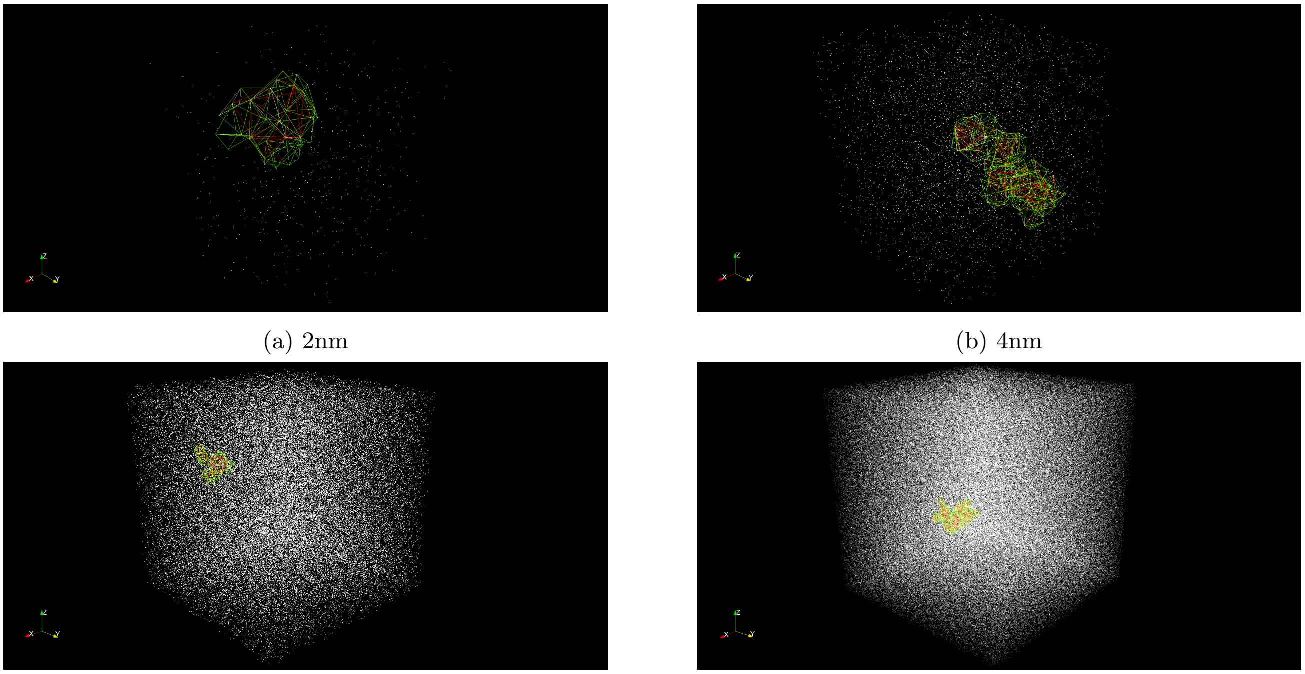

(a) 2nm

(b) 4nm

(c) 10nm

(d) 20nm

Figure S5: The results of the inverse analysis of a higher-order structure in each simulation cell size before expanding in sample 1. The shape is the strongest Delaunay complex in each cell size located in the point farthest from the diagonal in the persistent diagram. It can be seen that the size of the Delaunay complex occupying the entire cell is also reduced in the real cell size.

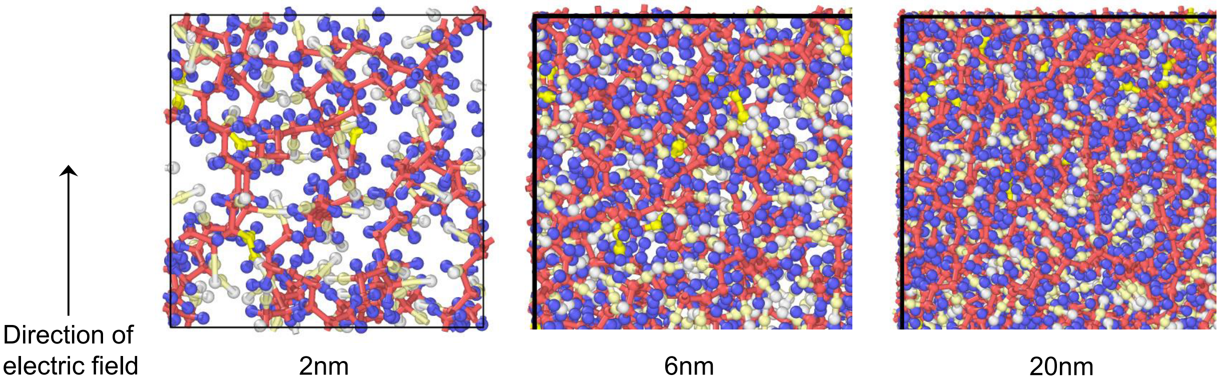

Figure S6: snapshot of orientation of polymer in each cell size in electric field environment in sample 1. The size of atoms are plotted in same in order to show the orientation of the main chain clearly.
